# Pan-cancer analysis of OSR2 with a focus on underlying mechanisms and therapeutic implications in lung adenocarcinoma

**DOI:** 10.3389/fimmu.2026.1769446

**Published:** 2026-04-24

**Authors:** Shijie Liu, Xuan Xiang, Siyu Liu, Wenbei Peng, Zihao Wang, Linlin Ye, Qiong Zhou

**Affiliations:** 1 Department of Respiratory and Critical Care Medicine, Union Hospital, Tongji Medical College, Huazhong University of Science and Technology, Wuhan, China; 2 Department of Respiratory and Critical Care Medicine, Chongqing Hospital, Union Hospital, Tongji Medical College, Huazhong University of Science and Technology, Chongqing, China

**Keywords:** OSR2, pan-cancer, tumor microenvironment, cancer-associated fibroblasts, prognostic biomarker, immunotherapy

## Abstract

**Background:**

The transcription factor Odd-skipped related 2 (OSR2) is involved in multiple physiological processes, yet its role in cancer pathogenesis remains largely undefined. Preliminary studies have suggested that OSR2 may contribute to the invasion and metastasis of several solid tumors. However, its function in the tumor microenvironment, its prognostic value and potential in predicting responses to immunotherapy across different cancer types are still inadequately explored and warrant a comprehensive systematic analysis.

**Methods:**

We performed an integrated pan-cancer analysis of OSR2 utilizing data from TCGA and GEO. Our analysis systematically evaluated OSR2 expression patterns, prognostic significance, and its correlations with tumor mutational burden (TMB), microsatellite instability (MSI), immune infiltration and immune checkpoint gene expression. Gene set enrichment analysis was employed for pathway enrichment analysis in both the pan-cancer bulk RNA-seq and LUAD single-cell transcriptomic data. The functional roles of OSR2 in LUAD were further validated through *in vitro* experiments.

**Results:**

OSR2 expression exhibited considerable heterogeneity across cancers, with elevated expression levels correlating with poor prognosis in several malignancies. Immune infiltration correlation and pan-cancer single-cell transcriptomics analysis revealed a strong association between OSR2 expression and cancer-associated fibroblasts (CAFs) in the tumor microenvironment (TME). Functional enrichment analysis suggested that OSR2 may promote tumor progression through induction of epithelial-mesenchymal transition (EMT). Furthermore, OSR2 knockdown attenuated the tumor-promoting effects of CAFs, resulting in suppressed proliferation and migration of LUAD cells *in vitro.*

**Conclusion:**

Collectively, our findings highlight the key role of OSR2 in tumor biology and tumor microenvironment regulation. OSR2 emerges as a promising prognostic biomarker and potential therapeutic target for human cancers; additionally, it may act as a reliable predictor of immunotherapy response in LUAD.

## Introduction

Cancer remains a major global threat to human health and life, with the annual incidence and mortality rates persistently increasing (1). According to recent data, there were approximately 18.5 million new cancer cases and 10.4 million deaths worldwide in 2023 (2). In recent years, advances in multimodal therapeutic strategies such as chemoradiotherapy, targeted therapy, and immunotherapy, have markedly improved the overall survival of cancer patients (3). However, despite these clinical breakthroughs, the molecular mechanisms underlying tumor initiation and progression exhibit considerable heterogeneity across different cancer types, and the regulatory pathways involved remain incompletely elucidated. Lung cancer, particularly lung adenocarcinoma (LUAD), represents one of the most fatal malignancies, with mortality rates that have risen significantly in recent years, posing a persistent threat to public health (4, 5).

Odd-skipped related transcription factor 2 (OSR2), a mammalian homolog of the Drosophila odd-skipped family, is a developmentally regulated transcription factor that modulates tissue patterning and organogenesis (6). The OSR2 gene is located on chromosome 8q22 and encode a protein product composed of 312 amino acids. OSR2 contains C2H2-type zinc finger domains, which influences cellular quiescence and proliferation through epigenetic regulation (7, 8). Physiologically, OSR2 is implicated in skeletal formation and tooth development (9–11). In human diseases, low expression of OSR2 is associated with recurrent spontaneous abortion (12), while elevated levels are associated with cancer progression such as muscle-invasive bladder cancer and endometrial carcinoma (13, 14). Nevertheless, the function of OSR2 in the tumor microenvironment (TME) remains poorly characterized, and its clinical relevance in lung adenocarcinoma has yet to be elucidated.

In this study, we performed an integrative analysis of OSR2 across multiple cancers utilizing publicly available datasets from The Cancer Genome Atlas (TCGA) and Gene Expression Omnibus (GEO). We systematically characterized OSR2 expression patterns, assessed its prognostic significance, and explored its correlations with tumor mutation burden (TMB), microsatellite instability (MSI), immune cell infiltration, and immune checkpoint gene expression in a pan-cancer context. Furthermore, we leveraged single-cell RNA sequencing data to delineate the role of OSR2 at single cell resolution in LUAD. Our results highlight the prognostic relevance of OSR2 in various tumor types, indicating its potential as a biomarker. In addition, the tumor-promoting role of OSR2 on LUAD was highlighted, which was further validated through *in vitro* experiments.

## Methods

### Pan-cancer data collection and processing

Transcriptomic and mutation data from The Cancer Genome Atlas (TCGA) dataset were downloaded using R package TCGAbiolinks. The UCSC Xena database (https://xenabrowser.net/) was utilized to obtain gene methylation data and cancer prognosis information. Specifically, Transcript per million (TPM) values were used for correlation and survival analyses, while raw count data were used for identifying differentially expressed genes and enrichment analysis. Additionally, the Cancer Cell Line Encyclopedia (CCLE) (https://portals.broadinstitute.org/ccle/) database was employed to analyze OSR2 expression across different cancer cell lines (15). Bulk transcriptomic data for NSCLC (Non-Small Cell Lung Cancer) were obtained from the GEO datasets GSE30219, GSE72094, GSE31210, GSE41271 and GSE135222, including gene expression and corresponding prognosis information. The single-cell RNA sequencing dataset GSE131907 was utilized. Additionally, immunohistochemical staining images of OSR2 in normal and tumor tissues were analyzed using data from the Human Protein Atlas (HPA) database (https://www.proteinatlas.org/) (16).

### Differential expression of OSR2 in tumors

The expression level of OSR2 between tumor and adjacent normal tissues across TCGA cancer types was assessed using the ‘gene_DE’ module of TIMER2 web platform (http://timer.cistrome.org/) (17). Furthermore, a paired comparative analysis was further conducted by examining OSR2 expression levels in matched tumor and normal tissue from the same patients within the TCGA.

### Analysis of survival and prognosis

The prognostic value of OSR2 for overall survival (OS), disease-specific survival (DSS), progression-free interval (PFI), and disease-free interval (DFI) was assessed using both Kaplan-Meier survival analysis and univariate Cox regression. We stratified all patients into high and low OSR2 expression groups based on the optimal cutoff value and compared their survival outcomes with Kaplan-Meier curves (18). The analyses were performed with the R packages survival and survminer. Subsequently, univariate Cox regression analysis was conducted using the survival and forestplot packages.

### Genomic alteration and mutational burden analyses

Pan-cancer analyses of the frequencies of genomic mutations, amplifications, and deep deletions were conducted with the cBioPortal for Cancer Genomics website (https://www.cbioportal.org/) (19). The R packages maftools and ComplexHeatmap were utilized to calculate the tumor mutation burden (TMB) and visualize the mutational landscape (20). Data on aneuploidy, neoantigen load, homologous recombination deficiency (HRD), and microsatellite instability (MSI) were sourced from previously published studies (21, 22). Correlation analysis between OSR2 expression and these genomic features was subsequently performed.

### DNA mismatch repair, stemness, and epigenetic modification analyses

Correlation analyses were performed between OSR2 mRNA expression and the expression of five mismatch repair (MMR) genes (23), four DNA methyltransferases (DNMTs) (24), and genes involved in post-transcriptional RNA modification (25, 26), with results visualized via heatmaps. We subsequently leveraged tumor cell stemness indices across different cancer types from prior studies (27, 28) to evaluate their association with OSR2 expression levels. Furthermore, methylation data from the UCSC database were employed to assess the relationship between OSR2 mRNA expression and methylation probe signals (29). The association between OSR2 promoter methylation and its gene expression was further illustrated using data from the GSCA platform (30).

### Immune infiltration and single-cell expression

Using the TIMER2.0 database, we obtained the relative infiltration abundance of immune and stromal cells across TCGA pan-cancer datasets. Subsequently, we performed correlation analysis between OSR2 mRNA expression levels and the infiltration abundance (17, 31), and visualized the results. The single-cell expression levels of OSR2 in pan-cancer were analyzed using the Tumor Immune Single-cell Hub (TISCH2) database (http://tisch.compbio.cn/home/). The expression data of OSR2 mRNA in different cell types of 75 datasets were downloaded and presented graphically using the R package ggplot2 (v3.5.2). In addition, violin plots showing the expression patterns of OSR2 in different cell types were obtained from the TISCH2 database (32).

### Gene set enrichment analysis and gene set variation analysis

The TCGA patients for each cancer type were divided into high and low expression groups based on the median expression level of OSR2. Gene Set Enrichment Analysis (GSEA) was then conducted using differentially expressed genes (DEGs) between these two groups. The Hallmark gene set was obtained from the Molecular Signatures Database (https://www.gsea-msigdb.org/gsea/msigdb). Normalized Enrichment Score (NES) and False Discovery Rate (FDR) were calculated using the DEGs utilizing the hallmark gene set, utilizing the R package clusterProfiler (33, 34). Additionally, we employed the GSVA R package to calculate enrichment scores for each sample in the pan-cancer datasets (35), and correlation analysis was performed between GSVA scores and OSR2 expression. All results were visualized using ggplot2 R package.

### Somatic mutation analysis in LUAD

LUAD samples were divided into high and low OSR2 expression groups based on the median OSR2 expression level. Somatic mutation landscapes were visualized using waterfall plots generated with the R package ComplexHeatmap, enabling a comprehensive summary of frequently mutated genes. Additionally, tumor mutation burden was compared between the two groups.

### Predictive analysis of immunotherapy response in LUAD

To assess the potential of OSR2 in predicting immunotherapy response, we computed the Tumor Immune Dysfunction and Exclusion (TIDE) score for LUAD samples from the TCGA database via the TIDE online platform (http://tide.dfci.harvard.edu/). Patients were stratified into high and low OSR2 expression groups for comparative analysis. In addition, Immunophenoscore (IPS) data for the TCGA-LUAD cohort were obtained from The Cancer Immunome Atlas (TCIA; https://tcia.at/home) and compared between OSR2-high and OSR2-low groups using t-tests (36). We also retrieved 13-step scores representing tumor immunogenicity and immune response processes from the Tumor Immunogenicity Profile (TIP) database. Differences in these scores between OSR2 expression groups were systematically evaluated in the TCGA-LUAD (37).

### Single-cell analysis of OSR2 in lung adenocarcinoma

The analysis utilized single-cell RNA-seq data from primary tumor tissue samples within the LUAD dataset GSE131907. Single-cell RNA-seq analysis was performed using the R package Seurat (v5.1.0) for graph-based visualization, cell type clustering, and dimensionality reduction. To investigate intercellular communication, we applied the R package CellChat (v1.1.3) to infer ligand-receptor interactions and construct cell-cell communication networks (38). Furthermore, potential ligand-receptor links between OSR2-high cancer-associated fibroblasts and epithelial (Epi) cells were specifically explored using the NicheNet. Cell-state transitions were analyzed with CytoTRACE (v0.3.3) and Monocle2 (v2.18.0) following default parameters (39, 40).

### Isolation of CAFs from malignant pleural effusions of LUAD patients

We isolated CAFs from malignant pleural effusions (MPE) using the following protocol. First, mononuclear cells isolated from malignant pleural effusions were resuspended in complete DMEM medium and plated uniformly in culture dishes. After 12 hours of incubation, non-adherent cells were discarded. Adherent cells were continued in culture and harvested with trypsin every two days. Following three cycles of incubation, CAFs purity was evaluated. For CAF purity assessment, approximately 1×10^5^ cells were subjected to flow cytometry analysis. CAFs were defined as the CD45^-^FAP^+^CD29^+^ cell population. Only CAFs with a purity greater than 90% were used for subsequent experiments. If the initial purity was below 90%, a fibroblast-specific isolation kit was employed for further purification.

### Cell transfection

The siRNA targeting OSR2 was synthesized by Tsingke Biotechnology Co., Ltd. (Beijing, China). Transfection of siRNAs was performed with Lipo3000(Invitrogen, Carlsbad, USA) according to the manufacturer’s protocol. The efficiency of transfection was validated using qRT-PCR. The sequences of the siRNAs are provided in **Supplementary File 1**.

### RNA extraction and quantitative real−time polymerase chain reaction

Total RNA was isolated from cell cultures with TRIzol reagent (Invitrogen, USA) in combination with the RNApure TissueCell Kit, and its concentration was quantified spectrophotometrically. Subsequent cDNA synthesis was performed with a dedicated reverse transcription kit. A Super SYBR Green Kit was used to perform qRT-PCR. Each sample was replicated at least three times, and the average value was used for data analysis. Primer sequences are provided in **Supplementary File 1**.

### Cell culture

The lung adenocarcinoma cell lines and CAFs were cultured in RPMI-1640 medium with 10% fetal bovine serum supplemented with 100 U/ml penicillin-streptomycin. All cell lines were incubated at 37 °C in 5% CO_2_. The cells for experiments in our study were passaged 10 times at most after the initial cell thawing.

### Conditioned medium production

CAFs were transfected with siRNA at a density of 1 × 10^6^ cells/mL and cultured in complete RPMI-1640 medium for 24 hours. This medium was then replaced with serum-free medium, and incubation continued for an additional 48 hours. Then the medium was centrifuged (2,000 × g, 10 min), and the supernatant was collected as CM. CM was used immediately or stored at -20 °C and thawed before application.

### Wound-healing assays

Cells (1 × 10^6^/well) were seeded in 6-well plates. When confluence was reached, a scratch was made using a 200 μL pipette tip. Cells were treated with medium containing CAF CM or siOSR2 CAF CM. Images of wound areas were captured at 0 and 24 h and analyzed with ImageJ.

### Cell proliferation assay

Cell proliferation was assessed using the Cell Counting Kit-8 (Beyotime, China). Briefly, 2000 A549 and H1299 lung cancer cells were seeded per well in 96-well plates and cultured for 5 days. Every 24 hours, 10 μL of CCK-8 reagent was added to each well, followed by incubation for 2 hours, and the absorbance was measured at 450 nm. For the colony formation assay, 1500 lung cancer cells were seeded per well in 6-well plates and cultured in 2 mL of conditioned medium. The medium was replaced every three days. Once formed, cells were fixed with 4% tissue cell fixative for 30 min, stained with 0.1% crystal violet for 20 min, air-dried, photographed, and the images documented.

### Transwell assay

To evaluate the ability to migrate and invade, transwell assays were conducted using transwell chambers (8 μm pore size; Corning) with and without Matrigel. In the upper chamber, a total of 2 × 10^4^ lung cancer cells were resuspended in 200 µL of serum-free medium. The corresponding CAFs (4 × 10^4^/well) were plated in the lower chamber, which was filled with complete medium containing 10% FBS. Cells were incubated at 37 °C for 24 h. After incubation, the migrated cells were fixed, stained, and counted in three randomly selected fields of view to evaluate migration and invasion.

### Statistical analysis

All statistical analyses were performed using R software (version 4.4.3). Differences between two groups were evaluated using either Student’s t-test or the Wilcoxon rank-sum test. Survival data were analyzed using the log-rank test. A two-sided *P* value < 0.05 was considered statistically significant. *P* values are presented as exact values in the text and figures unless otherwise indicated. (*P < 0.05, **P < 0.01, ***P < 0.001, ****P < 0.0001).

## Results

### Expression patterns and prognostic role of OSR2 in human cancers

**Figure 1:**
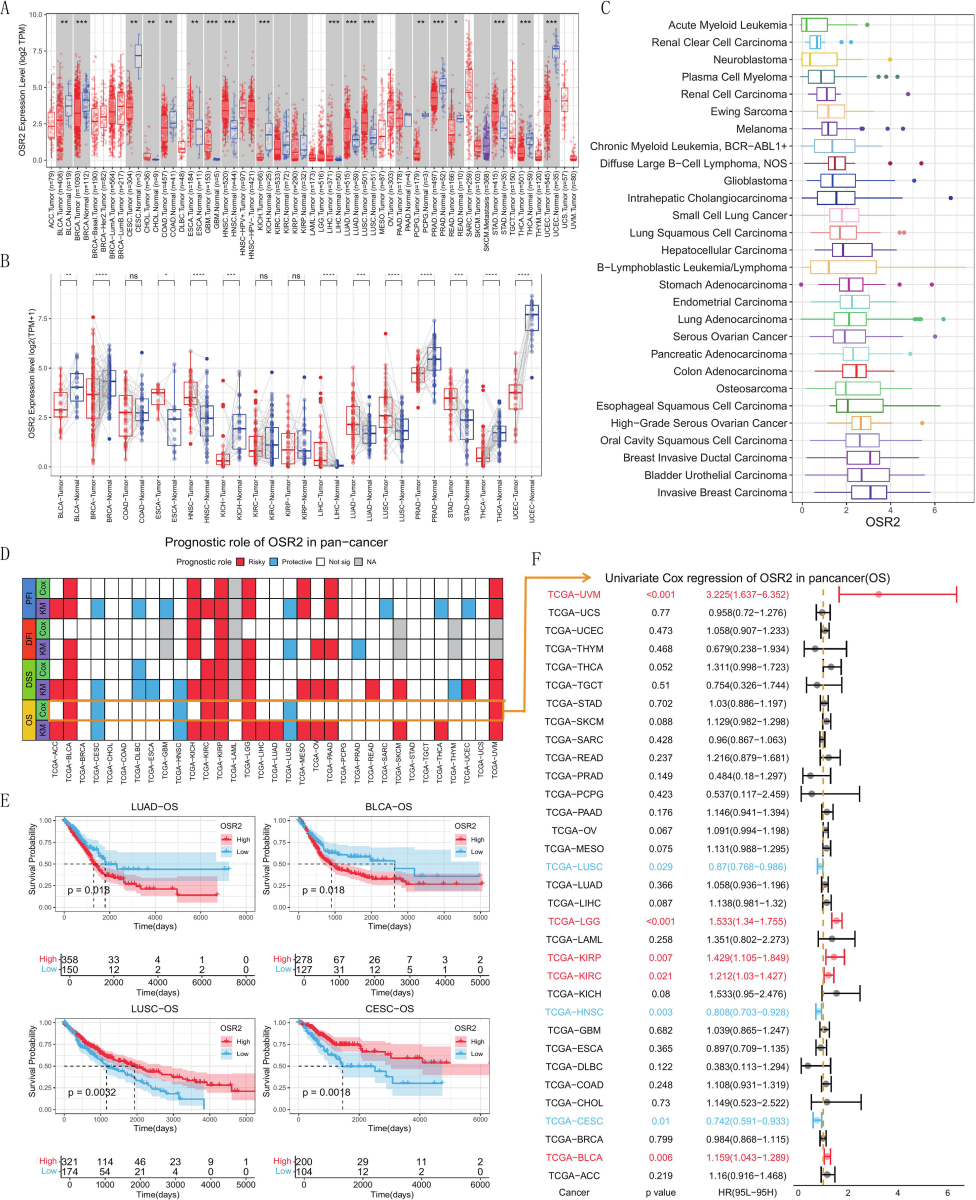
mRNA expression and prognosis of OSR2 in human cancers. **(A)** Differential expression of OSR2 across all TCGA tumors between the tumor and adjacent normal tissue. **(B)** Differential expression analysis between paired tumor and normal tissues. **(C)** OSR2 mRNA expression in cancer cell lines from the CCLE dataset. **(D)** Heatmap showing the correlation between OSR2 expression levels and four curated survival outcomes, including overall survival (OS), disease-specific survival (DSS), disease-free interval (DFI) and progression-free interval (PFI). Survival analysis was performed using a log rank (KM) test and univariate Cox regression, based on curated survival data from the TCGA database. Red boxes represent a risk factor, blue boxes represent a protective factor, white boxes represent the analyses are not significant, and gray boxes represent the data are not available. **(E)** Representative survival curves of prognostic analysis comparing OSR2 high and low patients in LUAD, BLCA, LUSC, and CESC. **(F)** Correlation of OSR2 expression with overall survival across pan-cancer. *P < 0.05, **P < 0.01, ***P < 0.001, ****P < 0.001.

We employed TIMER 2.0 website to explore the expression differences of OSR2 between tumor tissues and corresponding normal tissues in the TCGA database. As shown in **Figure 1A**, OSR2 mRNA expression was significantly upregulated in CHOL, ESCA, GBM, HNSC, LIHC, LUAD, LUSC, STAD and downregulated in BLCA, BRCA, CESC, COAD, KICH, PCPG, PRAD and UCEC. Consistent findings were observed in paired TCGA samples (**Figure 1B**). Furthermore, the HPA database also confirmed similar expression patterns of OSR2 in LUSC and LUAD (**Supplementary Figure 1A**). Analysis of the CCLE database revealed OSR2 expression across various tumor cell lines, with particularly high levels observed in BRCA and BLCA (**Figure 1C**). To investigate the prognostic impact of OSR2 in pan-cancer, we performed a comprehensive survival analysis. Heatmap clustering and survival analysis indicated that OSR2 served as a risk factor for patient prognosis in the 14 cancers including BLCA, KIRC, LUAD etc. Conversely, OSR2 was associated with a favorable prognosis in CESC, DLBC, HNSC, LUSC (**Figures 1D, E**). Univariate Cox regression analysis further demonstrated that high OSR2 expression was significantly correlated with worse OS in multiple cancers, such as BLCA, KIRC, and KIRP, and favorable in CESC, HNSC, LUSC (**Figure 1F**). Consistently, high OSR2 expression was associated with unfavorable outcomes in LUAD across several independent GEO cohorts (**Supplementary Figure 1B**).

### Genomic alteration and mutational burden analyses

**Figure 2:**
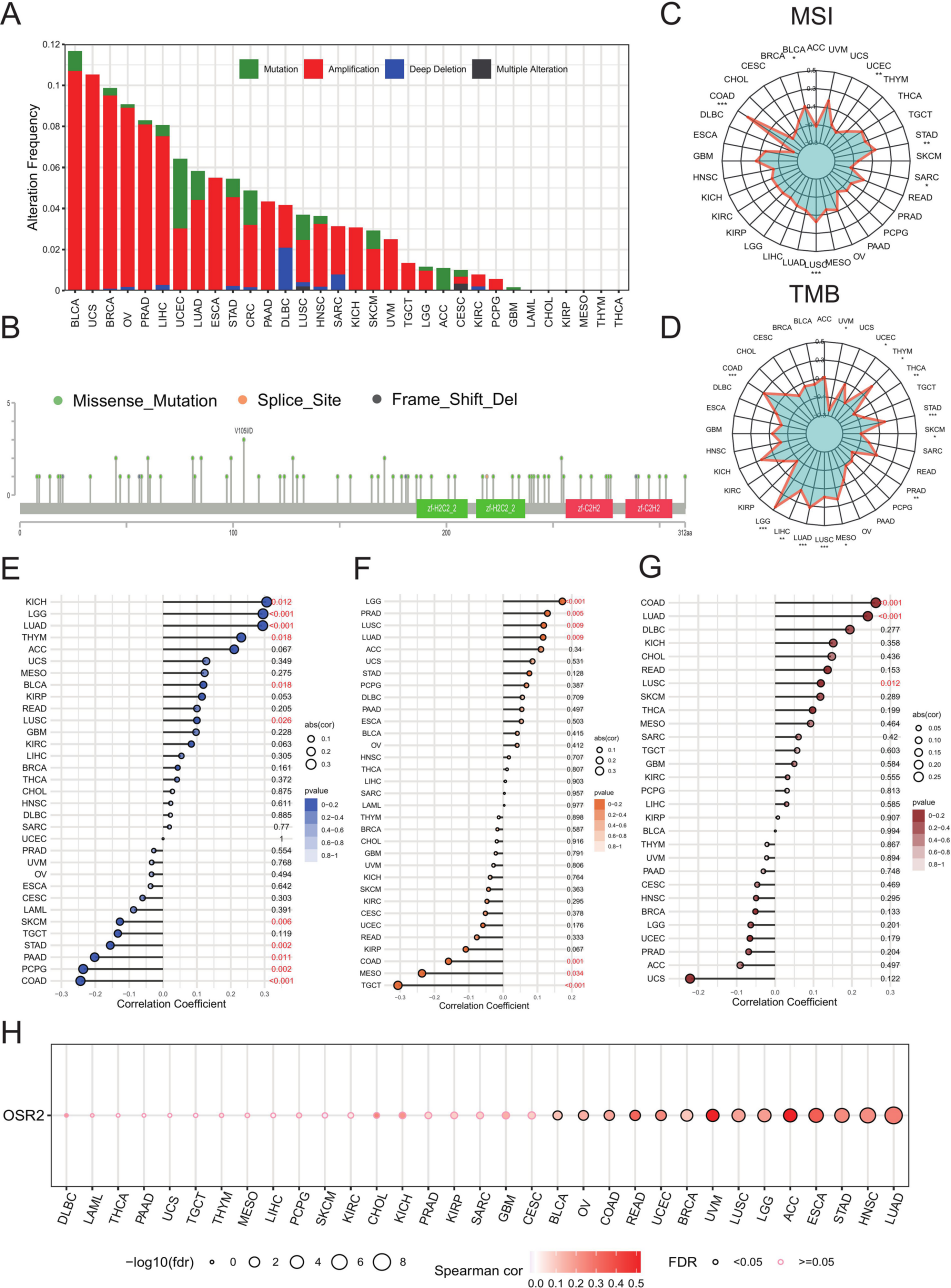
OSR2 expression is correlated with genomic instability. **(A)** Pan-cancer analyses of genomic changes in OSR2 in the TCGA database were conducted, including analyses of mutations, amplifications, and deep deletions. **(B)** The pan-cancer OSR2 SNV landscape, including missense, frameshift deletion, and splice site mutations. **(C, D)** Radar charts representing pan-cancer analyses of the link between OSR2 and both TMB **(C)** and MSI **(D)**. **(E, F, G)** Lollipop charts were used to visualize correlations between OSR2 mRNA levels and HRD **(E)**, aneuploidy **(F)** and neoantigens **(G)**, with dot sizes being proportional to sample sizes and dot color being proportional to p-values, with regular red font indicating that the cancer meets the p-value < 0.05 threshold. **(H)** Pan-cancer correlation between OSR2 expression and copy number variation (CNV) using Spearman’s correlation. *P < 0.05, **P < 0.01, ***P < 0.001.

Genomics strategies play a pivotal role in cancer research. To systematically investigate the genomic alterations of OSR2 across pan-cancer, we assessed its copy number variations (CNVs) and single nucleotide variations (SNVs). OSR2 amplification was primarily observed in BLCA, UCS, and BRCA, whereas deep deletions were common in DLBC. A higher frequency of SNVs was evident in UCEC and CRC (**Figures 2A, B**). Given that tumor mutational burden (TMB) and microsatellite instability (MSI) are well-established biomarkers for immunotherapy response and prognosis, we further evaluated the correlation between OSR2 expression and these indices. OSR2 expression showed a positive correlation with MSI in COAD, BLCA, STAD, and LUSC (**Figure 2C**). Moreover, OSR2 was positively associated with TMB in multiple cancer types, with the most significant correlations found in LGG, COAD, and LUAD (**Figure 2D**). Additionally, we also assessed the relationship of OSR2 with homologous recombination deficiency (HRD), aneuploidy, and neoantigen load (**Figures 2E–G**). Tumor-specific antigens, which are critical for eliciting anti-tumor immune responses, have been closely linked to prognostic outcomes across various cancers. To assess the association between OSR2 expression and copy number variation (CNV), we performed a pan-cancer analysis and identified the most significant correlation in LUAD (**Figure 2H**). Collectively, these findings suggest that OSR2 expression is significantly associated with genomic instability.

### DNA mismatch repair, stemness, and epigenetic modification analyses

**Figure 3:**
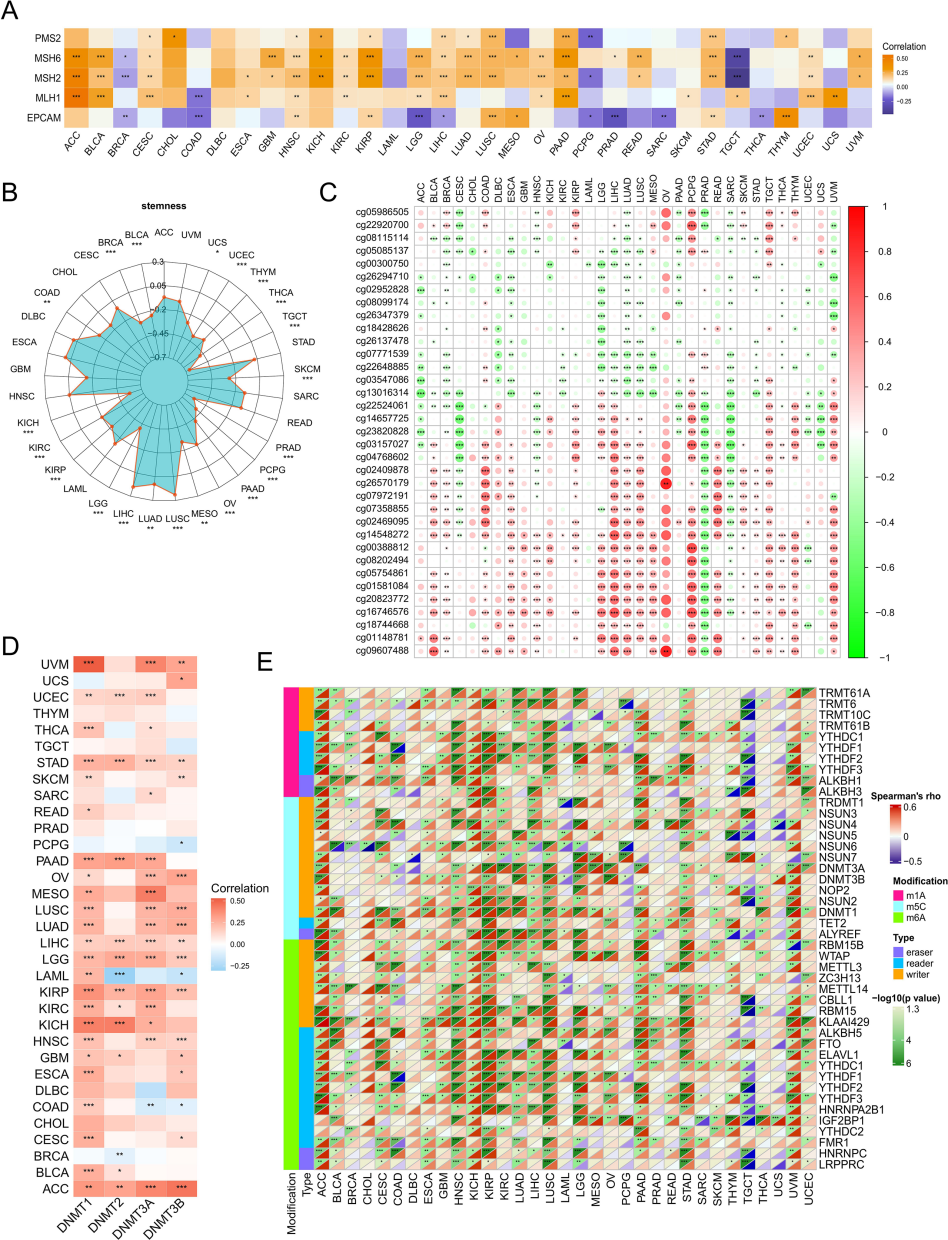
**(A)** Associations between OSR2 and five MMR genes across cancer types are presented in a heatmap. **(B)** Correlations between OSR2 mRNA levels and stemness are presented with a radar charts. **(C)** Correlations between OSR2 mRNA expression and DNA Methylation level. **(D)** Correlations between OSR2 and four DNMTS are presented in a heatmap. **(E)** Correlations between pan-cancer RNA modulations and OSR2 levels are presented in a heatmap. *P < 0.05, **P < 0.01, ***P < 0.001.

DNA damage response (DDR) is crucial for maintaining genomic integrity by detecting and eliminating aberrant chromosomal sequences and structures. Although cancer cells can exploit the mismatch repair (MMR) system to evade therapies and sustain self-renewal capacity, the relationship between OSR2 and MMR genes remains to be elucidated. Our analysis revealed a positive correlation between OSR2 expression and several key MMR genes across multiple cancer types, including ACC, BLCA, HNSC, KIRP, LUAD, and LUSC (**Figure 3A**). Interestingly, OSR2 exhibited a varied relationship with tumor cell stemness: it correlated negatively with stemness in numerous cancer types but showed a positive association in LIHC, LUAD and LUSC (**Figure 3B**). Epigenetic modifications play a pivotal role in tumorigenesis by modulating tumor cell proliferation, differentiation and progression. We first analyzed the correlation between OSR2 expression and DNA methylation levels. The specific CpG probes and their genomic locations used for this analysis are provided in **Supplementary Figure 2A**. Among all cancer types, LUAD exhibited the strongest negative correlation between OSR2 expression and overall DNA methylation (**Figure 3C**; **Supplementary Figure 2B**). Our analysis revealed a consistently positive correlation between OSR2 mRNA expression and DNMT genes across nearly all cancer types (**Figure 3D**). Additionally, OSR2 expression was positively associated with RNA modification genes in most tumors (**Figure 3E**), indicating its potential involvement in broader epigenetic and post-transcriptional regulatory networks.

### Immune infiltration and single-cell expression analysis

**Figure 4:**
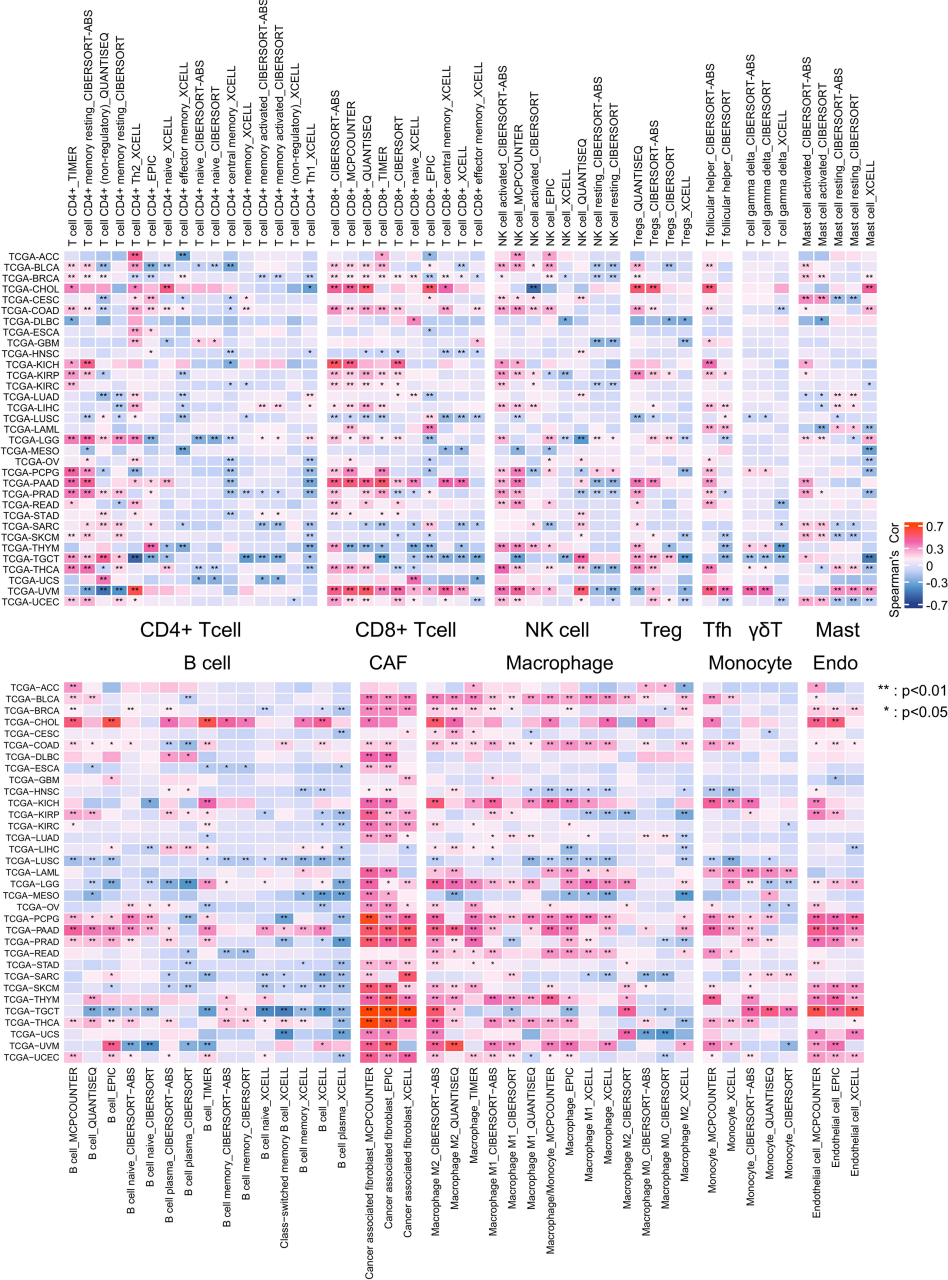
Correlation analysis between OSR2 expression and immune cell infiltration. Cluster heatmaps display the spearman correlation between OSR2 expressions and the degree of infiltration by CD4+T, CD8+T, NK, Treg, Tfh, γδT, mast, B, CAF, macrophage, monocyte, endothelial (Endo) infiltration. The red squares represent a positive correlation, while blue squares represent a negative correlation. *P < 0.05, **P < 0.01.

**Figure 5:**
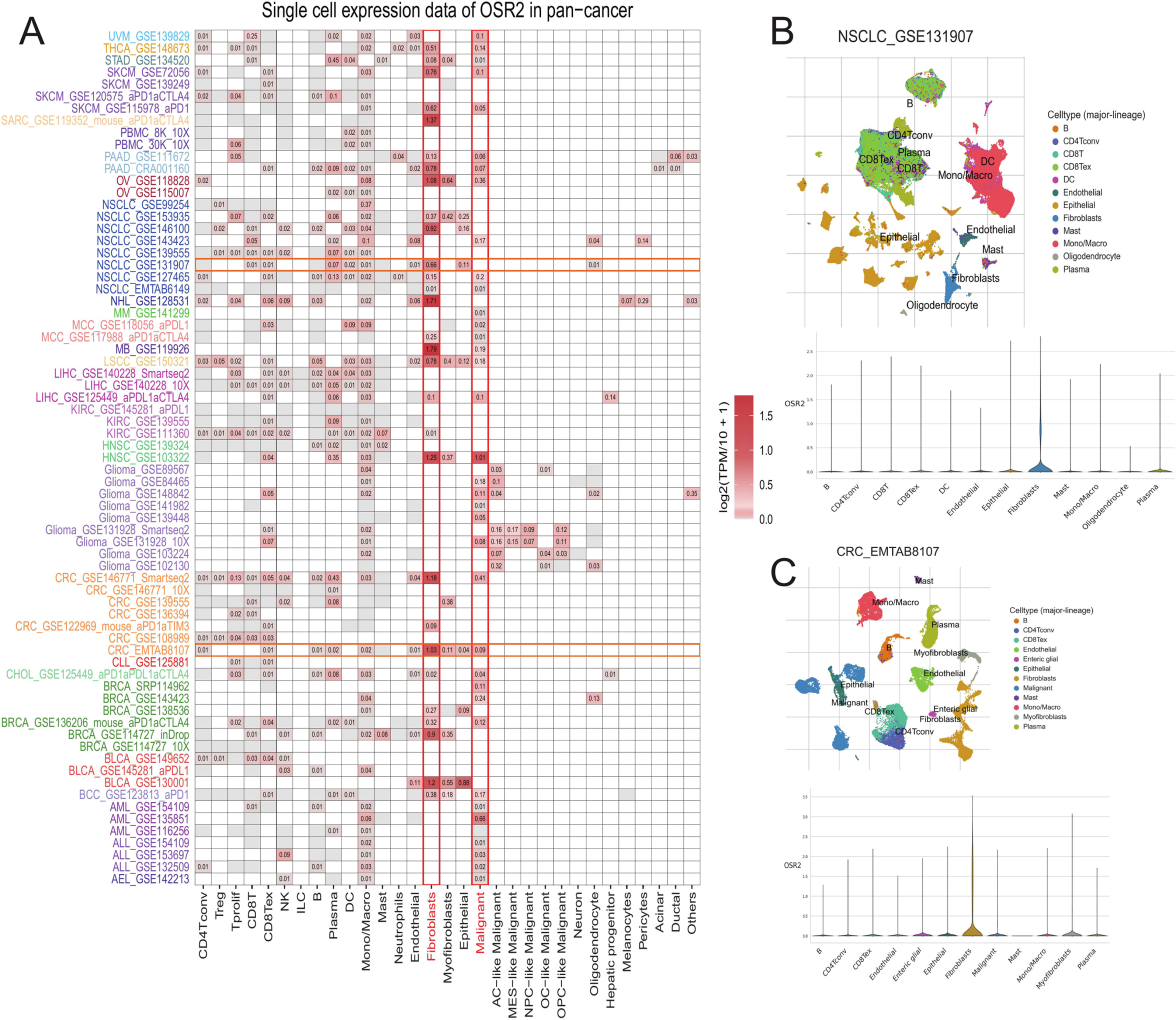
Single-cell expression analysis of OSR2 in tumor tissues. **(A)** Cluster heatmaps showing the mRNA levels of OSR2 in different cell types of tumor tissues. **(B, C)** Umap plots displaying the clustering of different cell types (upper panel) and OSR2 expression level (lower panel) in NSCLC and CRC tissues.

To investigate the association between OSR2 expression and immune cell infiltration in the TME across pan-cancer, we performed Spearman correlation analysis using data from the TIMER 2.0 database. As shown in **Figure 4**, OSR2 expression was negatively correlated with CD4+ T cells and B cells infiltration, but positively correlated with the abundance of macrophages, cancer-associated fibroblasts (CAFs), and endothelial cells across multiple cancer types. To further delineate the cellular expression pattern of OSR2, we analyzed single-cell RNA sequencing data from the TISCH2 database. At pan-cancer level, OSR2 expression was the highest in CAFs, followed by malignant epithelial cells, which was consistent with the correlation analysis (**Figure 5A**). We observed markedly elevated OSR2 expression in CAFs relative to all other cell types within the tumor microenvironments of LUAD and CRC (**Figures 5B, C**). We further performed Spearman correlation analysis to evaluate the associations between OSR2 expression and 47 immunomodulatory genes in pan-cancer. The analysis demonstrated that OSR2 was positively correlated with most immunomodulatory genes in CHOL, KICH, PAAD and UVM, whereas it showed a negative correlation with most in DLBC, LUSC (**Supplementary Figure 2C**).

### Enrichment analysis of OSR2 in human cancers

**Figure 6:**
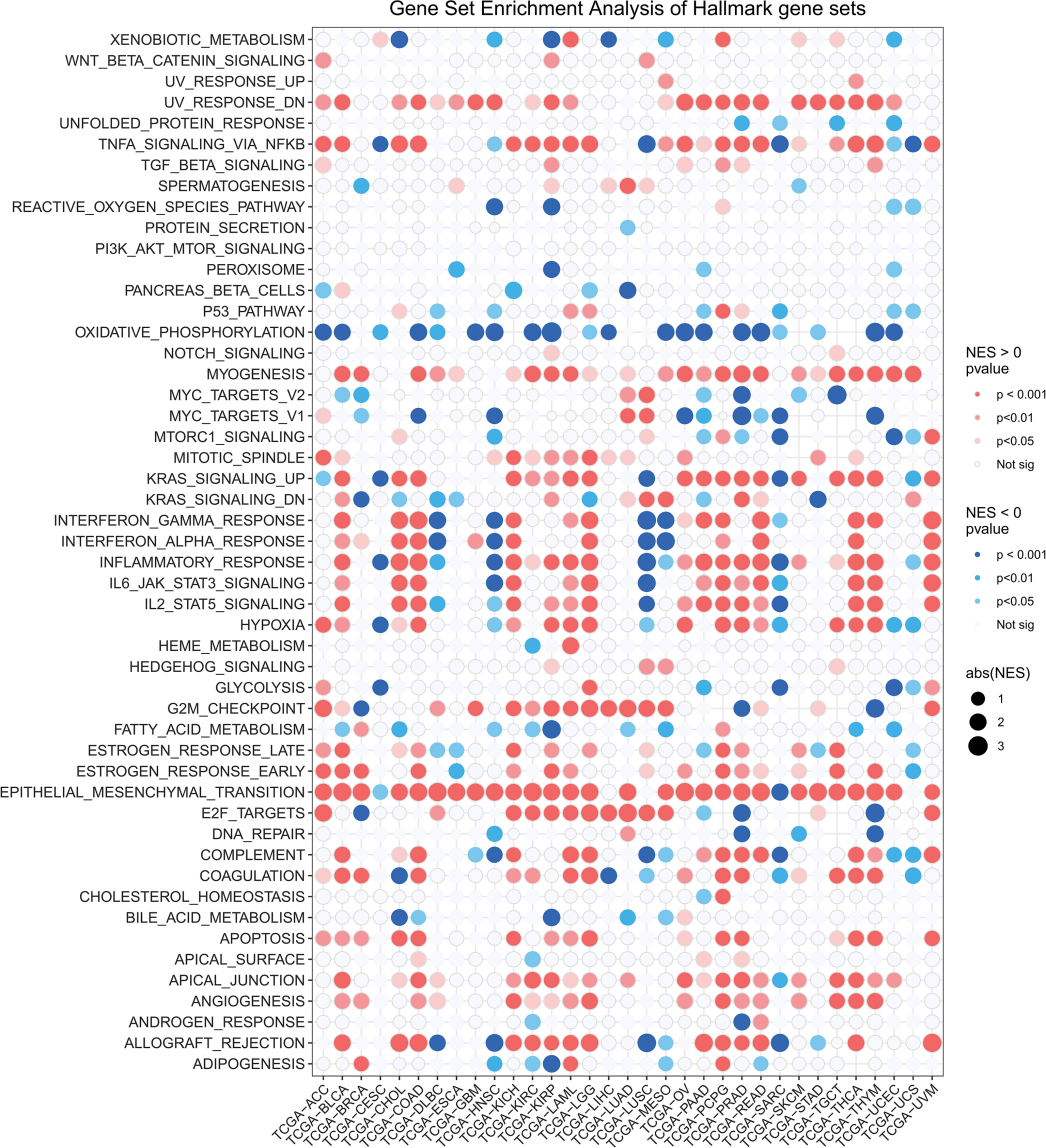
Potential function analysis of OSR2 in human cancers using GSEA. A bubble plot shows the results of GSEA between OSR2-high and -low tumor patients using hallmark gene signatures. The size of each circle represents the magnitude of normalized enrichment scores (NES), while the color codes represent the magnitude of the p and the sign of NES.

To explore the biological processes associated with OSR2 expression in cancers, we performed differential expression analysis by comparing the top and bottom 50% of OSR2 expression subgroup in each cancer type. Based on the differentially expressed genes (DEGs), we conducted Gene Set Enrichment Analysis (GSEA) across 33 cancer types to identify OSR2-associated cancer hallmarks. We found that immune-related pathways, including TNFA-signaling-via-NFKB, IFN-α response, IFN-γ response, and inflammatory response, were consistently enriched across multiple cancer types (**Figure 6**). Additionally, epithelial mesenchymal transition (EMT) and the G2M checkpoint pathways were also enriched in OSR2 high-expression group. These results suggest that OSR2 may be associated with both an immuno-inflammatory TME and enhanced tumor cell proliferation and migration. To further validate these findings, we then evaluated the correlation between OSR2 expression and GSVA scores of hallmark pathways across cancers. Our pan-cancer analysis revealed that OSR2 expression was associated with GSVA scores of inflammatory pathways and EMT pathways in multiple cancer types (**Supplementary Figure 3**).

### Association between OSR2 expression and the mutation landscape in LUAD

**Figure 7:**
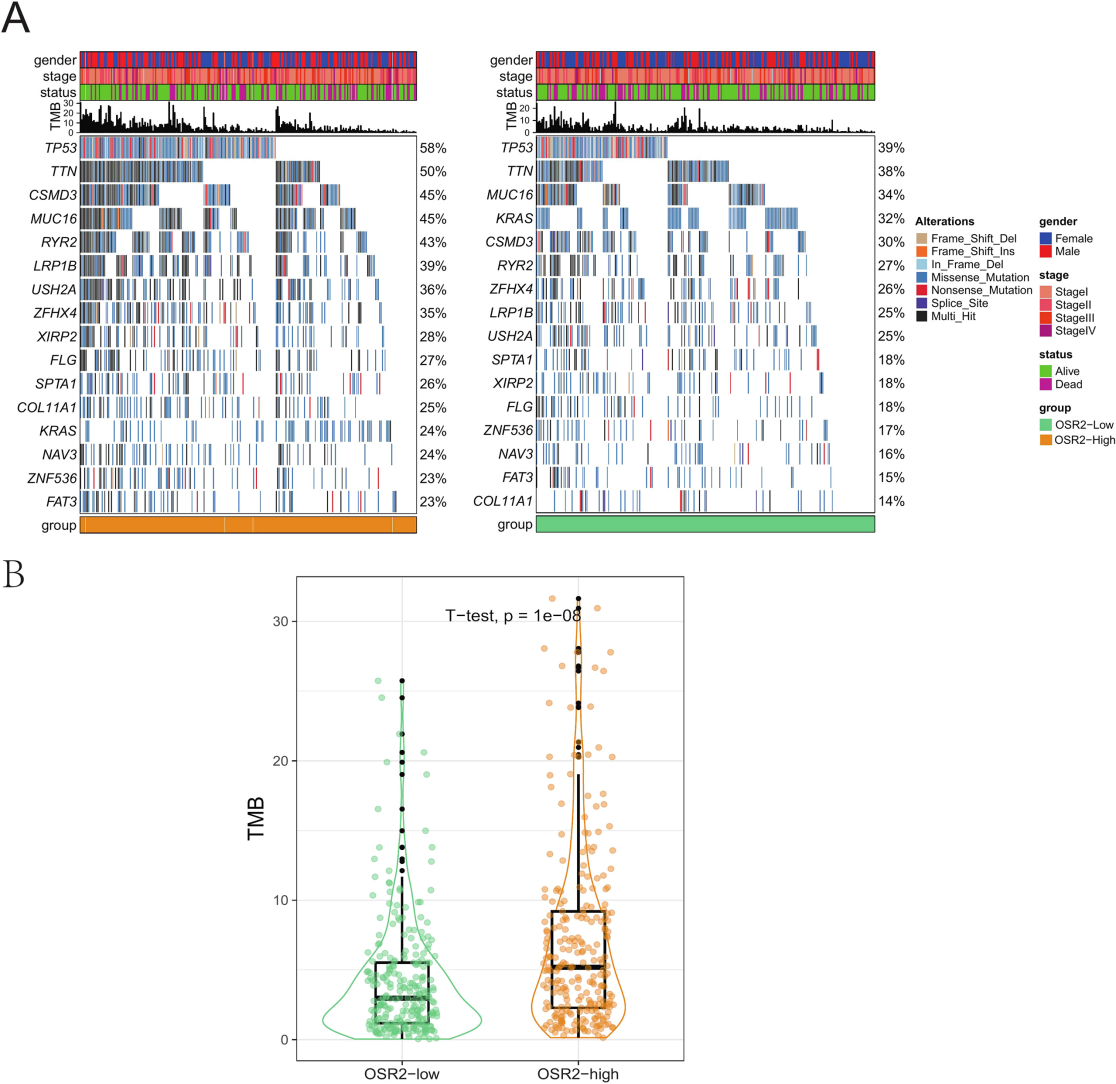
Mutation landscape in LUAD. **(A)** Mutation landscapes between high and low-expression of OSR2 in LUAD patients. **(B)** Tumor mutation burden between high and low-expression of OSR2 in LUAD patients.

Our previous pan-cancer analysis demonstrated OSR2’s connections to poor prognosis, genomic instability, and immune suppression. To further investigate the potential association between OSR2 expression and genomic instability in LUAD, we compared somatic mutations between tumors with high and low OSR2 expression. We found that while the top ten most frequently mutated genes were similar in both groups, their mutation frequencies differed markedly (**Figure 7A**). Moreover, OSR2 high expression group demonstrated a significantly elevated TMB compared to the low-expression group (**Figure 7B**, *p* < 0.001).

### OSR2 as a predictor of immunotherapy response in LUAD

**Figure 8:**
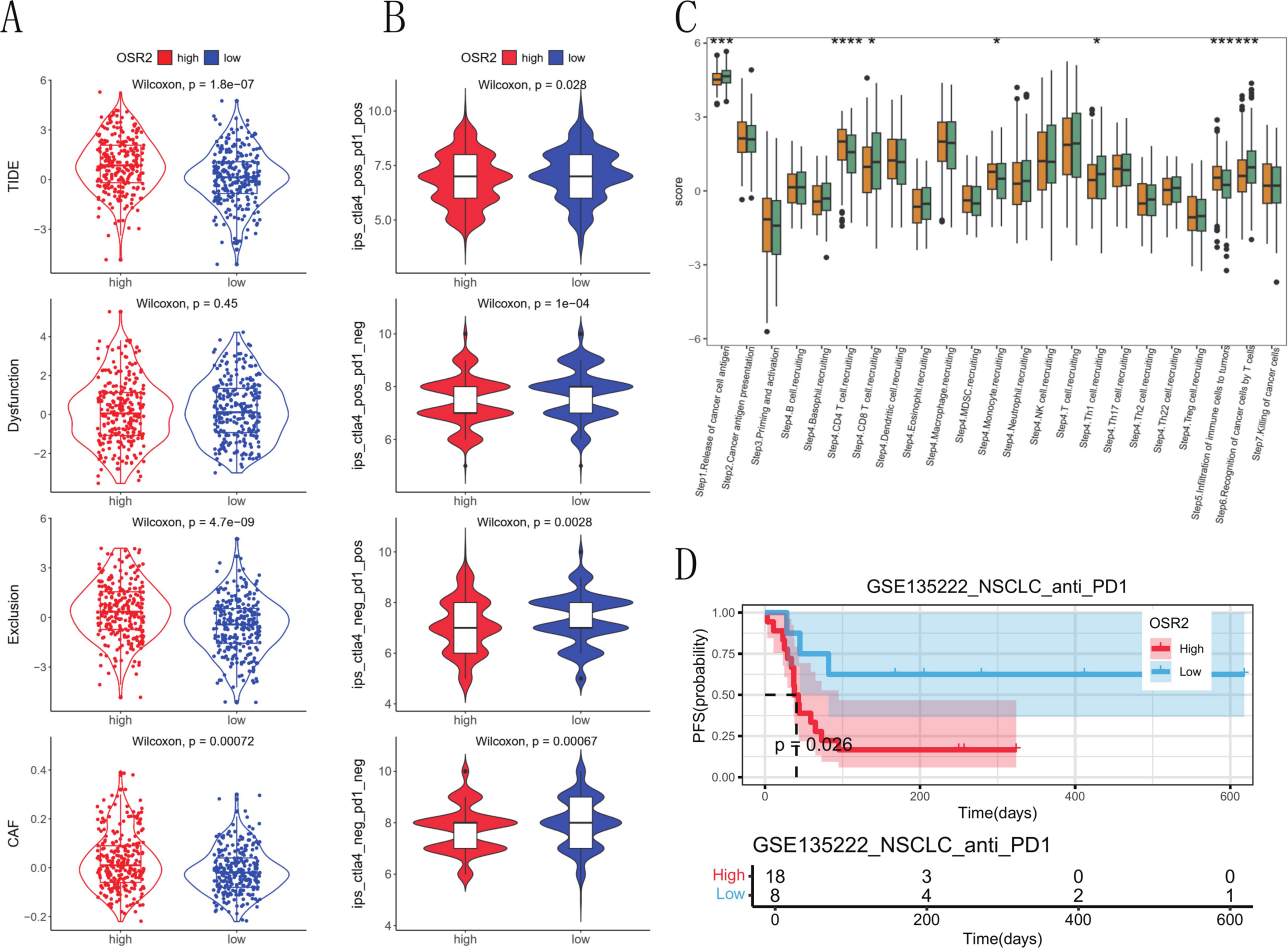
Influence of OSR2 expression on anti-tumor immunity and immunotherapy response. **(A)** The TIDE method calculates the TIDE scores for TCGA-LUAD datasets. **(B)** IPS for TCGA-LUAD datasets. **(C)** Boxplots display the correlation analysis of the cancer-immunity cycle in LUAD. **(D)** Predictive values of OSR2 expression on PFS of NSCLC patients in anti-PD-1 immunotherapy. *P < 0.05, **P < 0.01, ***P < 0.001.

Based on our single-cell analysis indicating predominant OSR2 expression in CAFs, we sought to evaluate the potential of OSR2 as a predictor of immunotherapy response in LUAD. Using the Tumor Immune Dysfunction and Exclusion (TIDE) scores and Immunophenoscores (IPS) as predictive metrics, we demonstrated that the OSR2-high group exhibited significantly elevated TIDE scores and reduced IPS scores, suggesting a potentially diminished benefit from immunotherapy in these patients (**Figures 8A, B**). We further assessed the activity scores of cancer-immunity cycles using data from the TIP database. As shown in **Figure 8C**, the OSR2-high group exhibited significantly suppressed activity in multiple key processes, including cancer cell antigen (Step1), CD8^+^ T cell recruitment (Step4), and T cell-mediated tumor cell recognition (Step6). Subsequently, we analyzed the prognostic value of OSR2 expression in the context of immune checkpoint inhibitor therapy. Survival analysis showed that among NSCLC patients treated with anti-PD-1 therapy, those with low OSR2 expression exhibited significantly prolonged progression-free survival (PFS) compared to those with high OSR2 expression (**Figure 8D**).

### Single-cell analysis of OSR2 in LUAD

**Figure 9:**
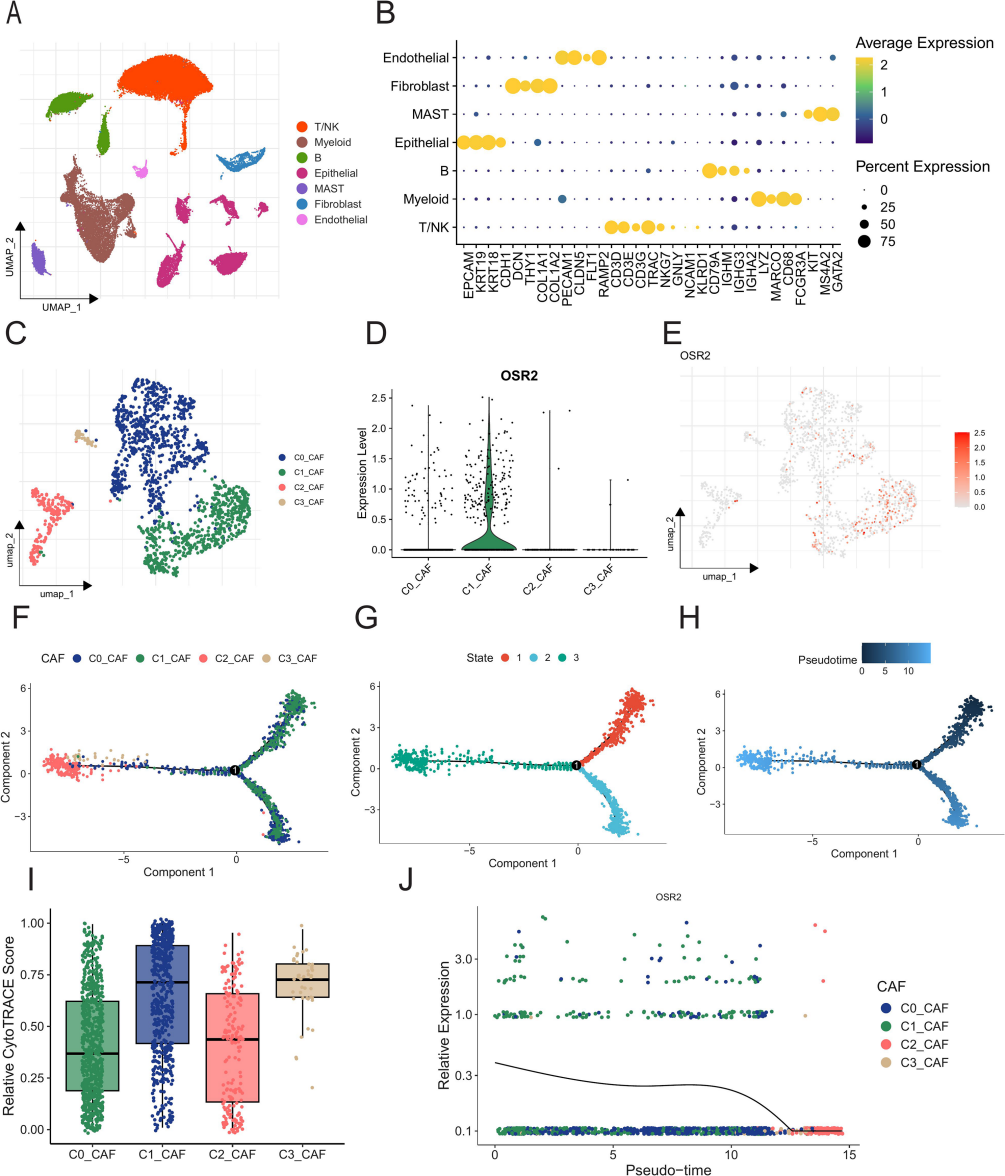
Single-cell RNA-seq data analysis and cell annotation. **(A)**UMAP plot of the profiled single cell. **(B)** Dot plots showing average expression of known markers in indicated cell types. **(C)** UMAP visualization of fibroblasts composition, with colors indicating different cell types. **(D, E)** Violin and feature plots illustrating the expression of OSR2 gene in each CAFs subgroup. **(F–H)** The arrangement of CAFs subtypes along pseudotimes within the two-dimensional state space as determined by Monocle2. **(I)** Boxplot displaying the distribution of CytoTRACE score in CAF subgroups. **(J)** The expression of OSR2 along pseudotimes.

Based on single-cell RNA sequencing data of 13 primary LUAD samples from GSE131907, we performed an integrated analysis using the Seurat package. Following standard preprocessing, dimensionality reduction, and clustering, we identified 11 distinct cell clusters, which were subsequently annotated as seven major cell types: T/NK cells (marked by CD3D, CD3E), B cells (CD79A, MS4A1), myeloid cells (LYZ, CD68), fibroblasts (DCN, COL1A1), endothelial cells (CLDN5, RAMP2), and epithelial cells (KRT18, KRT19) (**Figures 9A, B**; **Supplementary Figure 4A**). As OSR2 was mainly expressed in CAFs, we further divided fibroblasts into several subtypes and clustered them into 4 subtypes (**Figure 9C**). Violin and feature plots demonstrated that OSR2 was highly and specifically expressed in the C1_CAF subtype (**Figures 9D, E**). Pathway enrichment analysis indicated that the OSR2-high C1_CAF subtype was significantly enriched in processes such as the inflammatory response and epithelial-mesenchymal transition (EMT) compared to other fibroblast subtypes, consistent with our prior pan-cancer findings (**Supplementary Figures 4B–D**). To investigate the differentiation relationships among fibroblast subtypes, we performed pseudotime trajectory analysis using Monocle2 and assessed cellular stemness potential with CytoTRACE, based on the gene expression profile of each individual cell. The results demonstrated that the C1_CAF subtype exhibited higher stemness properties than C0_CAF, as reflected by a less differentiated state. Pseudotime trajectory analysis positioned C1_CAF subtype at the starting point of the differentiation trajectory (**Figures 9F–I**). Furthermore, OSR2 expression progressively decreased along the pseudotime axis, suggesting a potential role in early fibroblast differentiation within the LUAD microenvironment (**Figure 9J**).

### Inference of cell–cell communications in TME

**Figure 10:**
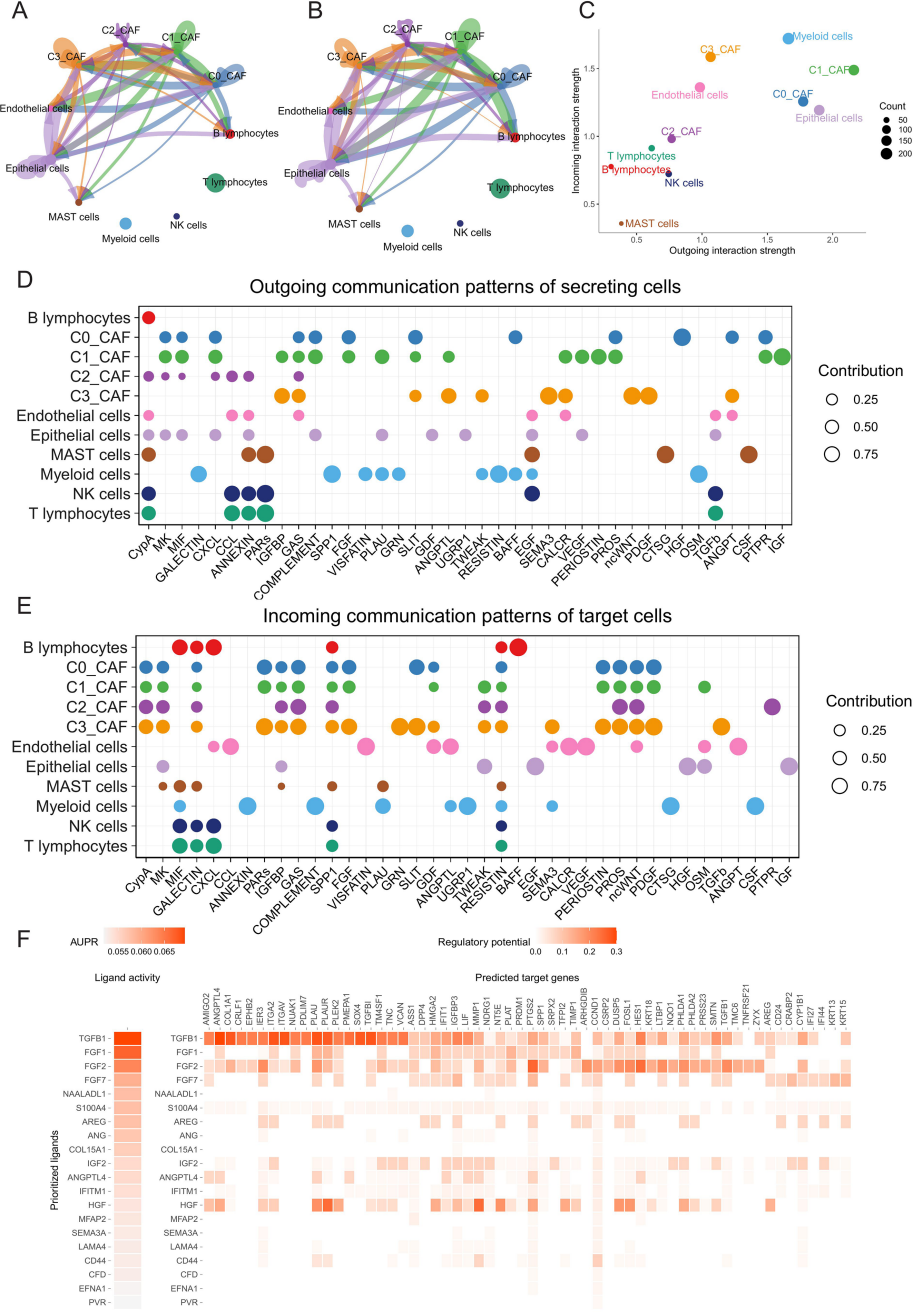
Inference of cell–cell communications in TME. **(A)** Differential interaction quantity and **(B)** strength of cell-cell communications analyzed by CellChat. **(C)** Illustration of the incoming and outgoing interaction strengths for each of the cell types. **(D)** The outgoing signaling pathways of each cell type. **(E)** The incoming signaling pathways of each cell type. **(F)** Heatmaps (NicheNet) showing ligand activity of top-ranking ligands from OSR2-high C1_CAF (left) and their regulatory potential on predicted target genes in epithelial cells (right).

We used the CellChat tool to quantitatively infer intercellular communication networks from scRNA-seq data and investigate its relevance to pro-tumoregenic processes. First, we analyzed the number and strength of interactions between CAFs and epithelial cells in LUAD samples. The majority of interactions were observed among C0_CAF, C1_CAF and epithelial cells. When interaction strength was taken into account, C1_CAF showed particularly strong communication with epithelial cells (**Figures 10A, B**). The inferred incoming and outcoming interaction patterns revealed that C1_CAF serves as the primary CAF subtype expressing ligands and receptors actively engaged in the cellular interactions (**Figure 10C**). To reveal the signaling pathways contributing to the complex intercellular communications, we quantified the outgoing and incoming interaction strength of each pathway (**Figures 10D, E**). Additionally, we applied NicheNet to predict ligand-receptor-target gene interactions between malignant epithelial cells and C1_CAFs. The heatmap highlighted the top 20 most active ligands derived from C1_CAFs that influence malignant epithelial (**Figure 10F**). Among these, ligands such as TGFB1, FGF1 and FGF2 were highly active. We also investigated the potential target genes for the top20 ligands with the highest activity (**Figure 10F**).

### Cell proliferation and migration

**Figure 11:**
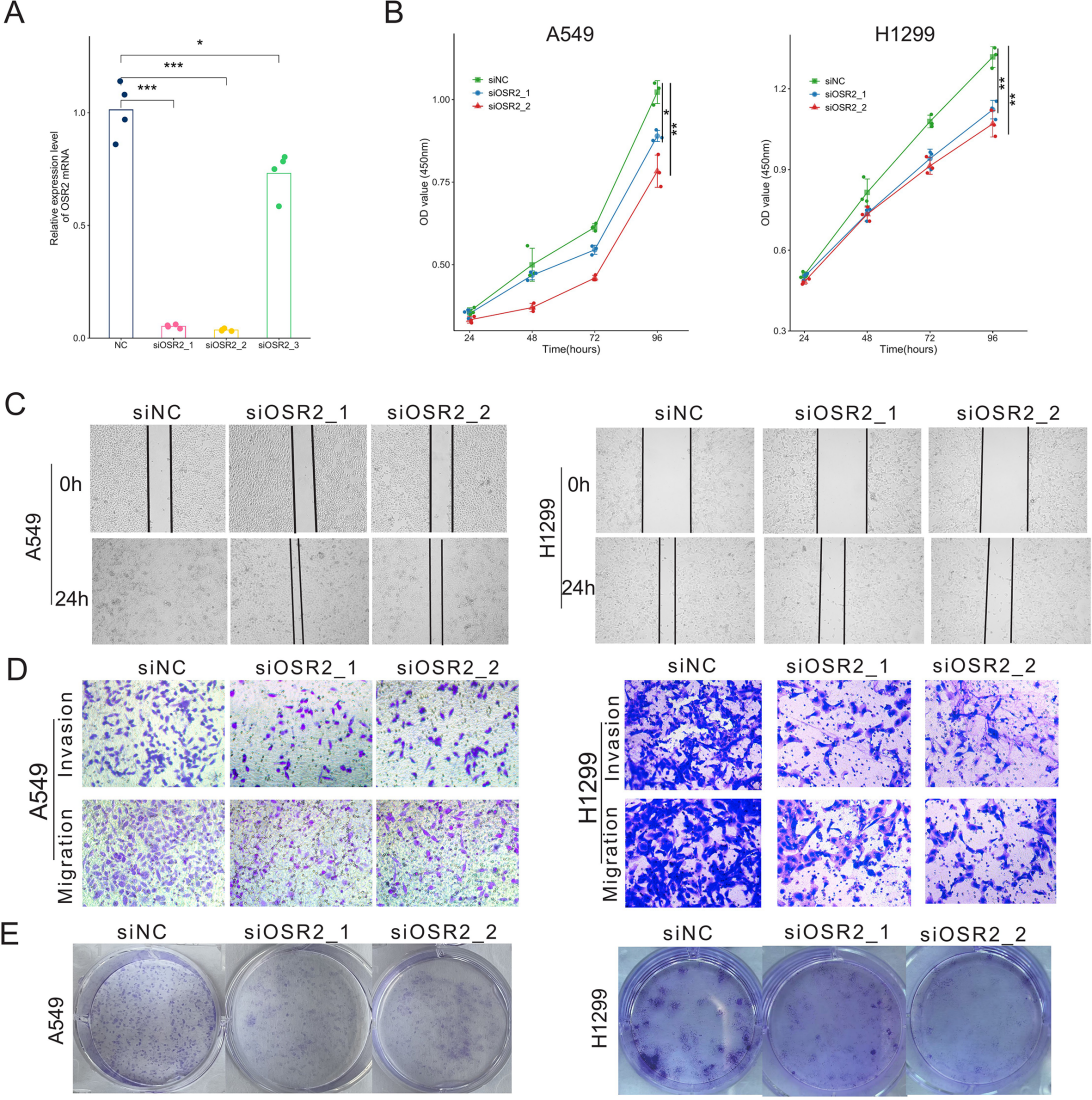
OSR2 knockdown in CAFs partially reverse proliferation, colony formation, migration, and invasion of LUAD cells. **(A)** RT–qPCR results of OSR2 siRNAs on CAFs. **(B)** Proliferation assay measuring OD values of tumor cells in conditioned medium (CM) from CAF cell lines, with three biological replicates per group. **(C)** Wound healing assays for A549 and H1299 cells treated with CAF CM. **(D)** Transwell migration and invasion assays of A549 and H1299 cells treated with CAFs. **(E)** Colony formation assays A549 and H1299 cells following the same treatments as in **(B)**. *P < 0.05, **P < 0.01, ***P < 0.001.

The purity of cultured CAFs was assessed by flow cytometry prior to downstream experiments. Only cell preparations with a CAF purity greater than 90% were used for subsequent analyses. The detailed gating strategy is shown in **Supplementary Figure 4E**. To further investigate the role of OSR2 in tumor progression, we knocked down its expression in CAFs and evaluated the resulting effects on tumor cells. RT-qPCR revealed that OSR2 siRNA1 and siRNA2 effectively decreased the expression of OSR2 mRNA in CAFs (**Figure 11A**). Therefore, siRNA1 and siRNA2 were chosen for further experiments. Conditioned media (CM) were collected from CAFs transfected with OSR2-targeting siRNA (siOSR2-CAF) or negative control siRNA (siNC-CAF). These CM were then applied to treat the LUAD cell lines A549 and H1299 for functional analysis. The results demonstrated that CM derived from OSR2-knockdown CAFs significantly suppressed the proliferation, migration and colony formation of lung cancer cells compared to control CM (**Figures 11B–E**), indicating a strong pro-tumor activity of OSR2-high CAFs.

## Discussion

Multi-omics technologies have become instrumental in cancer research for elucidating molecular features and identifying key driver genes, thereby facilitating the discovery of novel therapeutic targets (41). In this study, we conducted a comprehensive pan-cancer analysis to evaluate the expression landscape and prognostic relevance of OSR2 across 33 cancer types. Our analysis revealed that OSR2 expression varied substantially among cancers, with elevated expression frequently associated with unfavorable prognosis in multiple cancers, suggesting its potential utility as a prognostic biomarker.

Previous studies have established the importance of OSR2 in early development processes (9–11). In recent years, its tumor-promoting roles have been documented in specific cancers (13, 14). Based on these previous researches, we systematically evaluate OSR2 across broad spectrum of tumors and demonstrated that it functions as a risk factor in cancers such as ACC, BLCA, KIRC, KIRP, and LUAD, while serving as a protective factor in CESC, HNSC, and LUSC. Numerous studies have reported that a single gene can exert divergent or even opposing effects across different cancer types, which may be attributed to distinct tumor microenvironments and the activation of context-dependent molecular pathways (42–44). In line with this, the opposing roles of OSR2 across cancers likely reflect its context-dependent transcriptional activity, where its downstream targets and regulatory networks may differ fundamentally between tumor types. This functional duality underscores the complexity and heterogeneity of OSR2 in cancer biology.

The immune composition of the TME critically influences tumor behavior and therapeutic response (45, 46). Our pan-cancer analysis indicated that OSR2 expression correlates with the infiltration of immune cells and stromal cells. Specifically, OSR2 levels were negatively associated with CD4^+^ T cells and B cells, which were known to support anti-tumor immunity (47, 48). While OSR2 levels were positively correlated with macrophages, endothelial cells, and CAFs, with the strongest association observed in CAFs. As CAFs are known to promote tumor growth, therapy resistance, and tumor invasion through mechanisms such as induction of epithelial-mesenchymal-transition (EMT) (49, 50). Thus, these findings imply that high OSR2 expression may foster an immunosuppressive TME characterized by diminished anti-tumor immune activity and enhanced pro-tumor stromal engagement. This notion is further supported by the significant positive correlations between OSR2 and various immune-inhibitory molecules across multiple cancer types in the pan-cancer context. These results provide further support for the role of OSR2 in mediating immunosuppression in the TME and influencing the efficacy of immunotherapy.

In our analysis, the GSEA results indicated that OSR2 was significantly associated with immune-related pathways and EMT pathway. Previous studies have demonstrated that the inflammatory microenvironment is a critical driver of lung adenocarcinoma progression (51). Specifically, high OSR2 expression was correlated with enrichment in TNF-α signaling via NF-KB, IFN-α/γ response, and inflammatory response across multiple cancer types (**Figure 6**), indicating its pivotal role in shaping an inflammatory and immunomodulatory TME. Given that EMT is a key driver of tumor invasion and metastasis (52, 53), these findings provide mechanistic insight into how OSR2 may contribute to both immune evasion and malignant progression.

Focusing on LUAD, we compared the mutational landscape between OSR2-high and OSR2-low tumors and found that the OSR2-high group exhibited a significantly higher TMB (*p* < 0.001). As high TMB often reflects genomic instability and has been correlated with aggressive tumor behaviors in LUAD (54, 55), this findings reinforce the potential role of OSR2 as an indicator of tumor aggressiveness. Given the established efficacy of immunotherapy in advanced LUAD (56, 57), we further evaluated the relationship between OSR2 expression and immunotherapy response. The TIDE score estimates the likelihood of tumor immune escape by modeling T cell dysfunction and exclusion, thereby predicting immune checkpoint inhibitor response. And Immunophenoscore (IPS) assesses tumor immunogenicity by integrating the expression of immunomodulatory genes, immune cell infiltration, and neoantigen. Using the two well-validated predictors, we demonstrated that the OSR2-high group displayed significantly higher TIDE scores and lower IPS values, suggesting that elevated OSR2 expression may be associated with poorer response to immunotherapy in LUAD.

Accumulating evidence highlights the multifaceted role of CAFs in promoting tumor progression, metastasis, and therapy resistance (58, 59). Our single-cell RNA sequencing analysis revealed an OSR2-high CAF subtype that was associated with pro-tumorigenic pathways and active intercellular communication. Functional validation using conditioned medium from OSR2-knockdown CAFs demonstrated a significant reduction in the ability to promote tumor cell proliferation, migration and colony formation in LUAD cells. These findings collectively identify OSR2 as a key functional regulator that contributes to the pro-tumorigenic phenotype of CAFs in LUAD.

Despite the strengths of our pan-cancer analysis, there were some limitations should be acknowledged. First, our analysis relied on publicly available databases. Although these databases offer large-scale datasets, they are subject to potential limitations such as data heterogeneity and batch effects. Second, while our results indicate that OSR2 may serve as a pan-cancer prognostic biomarker and potential therapeutic target, especially with relevance to immunotherapy in LUAD, these findings still require further validation in larger and independent clinical cohorts. Third, although our functional studies highlighted OSR2-high CAFs as key promoters of LUAD progression, the specific secretory factors downstream of OSR2 and their detailed mechanisms in regulating EMT in tumor cells remain to be fully elucidated.

## Conclusion

In summary, our study demonstrates that high OSR2 expression is associated with poorer prognosis in most cancer types examined, with the exception of CESC, DLBC, HNSC, and LUSC. Importantly, we identified CAFs as the primary source of OSR2 within the TME and revealed its pro-tumorigenic function in promoting cell proliferation and inducing EMT. These findings collectively suggest OSR2 as a promising prognostic biomarker and a potential therapeutic target. Further investigation into its mechanistic roles is therefore warranted and may pave the way for discovering novel therapeutic targets for cancer treatment.

## Data Availability

The original contributions presented in the study are included in the article/**Supplementary Material**. Further inquiries can be directed to the corresponding author.
