# Supplementary material for: Pan-cancer analysis of OSR2 with a focus on underlying mechanisms and therapeutic implications in lung adenocarcinoma
Source: Front Immunol. 2026 Apr 24;17:1769446. doi: 10.3389/fimmu.2026.1769446 (PMC13152841; doi:10.3389/fimmu.2026.1769446)
Supplement: Supplementary file 2 [file Table1.docx]

qPCR primer

OSR2

OSR2-F1: ACACATGCAGGAATCTCCACACA

OSR2-R1: TTTCGCCTGAACACTTTGCCG

GAPDH：

GAPDH-F1: GGAGTCAACGGATTTGGTCG

GAPDH-R1: GGAATCATATTGGAACATGTAAACC

siRNA sequence

siOSR2_1: Forward, GGCAAAGUGUUCAGGCGAATT

siOSR2_1: Reverse, UUCGCCUGAACACUUUGCCTT

siOSR2_2: Forward, CGGCAGACACUUUACCAAATT

siOSR2_2: Reverse, UUUGGUAAAGUGUCUGCCGTT

siOSR2_3: Forward, CUAGAACUCUAGCAGUUCATT

siOSR2_3: Reverse, UGAACUGCUAGAGUUCUAGTT

siRNA NC: Forward, UUCUCCGAACGUGUCACGUTT

siRNA NC: Reverse, ACGUGACACGUUCGGAGAATT
