# Supplementary material for: Pan-cancer analysis of OSR2 with a focus on underlying mechanisms and therapeutic implications in lung adenocarcinoma
Source: Front Immunol. 2026 Apr 24;17:1769446. doi: 10.3389/fimmu.2026.1769446 (PMC13152841; doi:10.3389/fimmu.2026.1769446)
Supplement: Supplementary file 3 [file Table2.docx]

Supplementary Figure


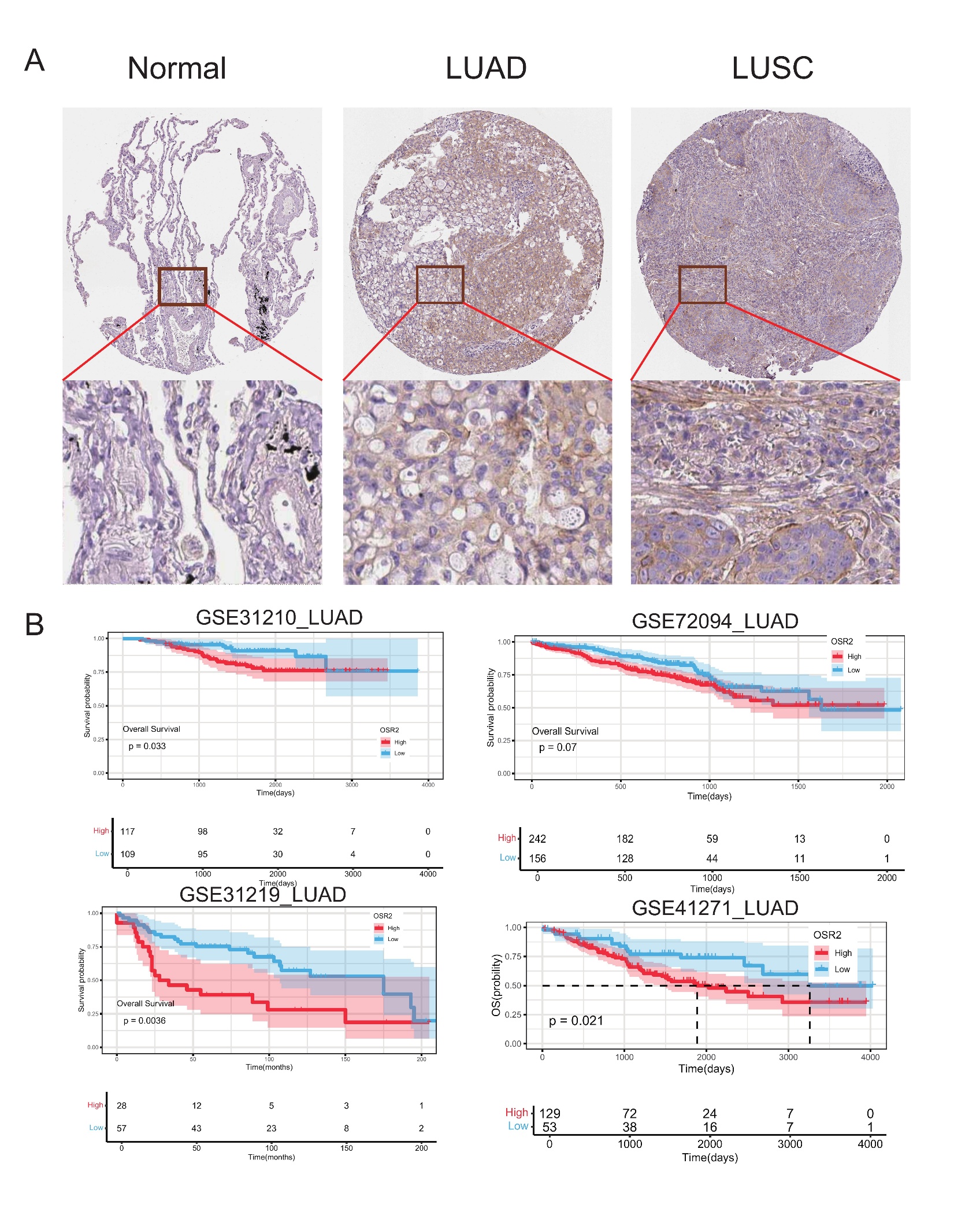


Supplementary Fig.1 (A) Representative IHC images of OSR2 expression in normal lung, LUAD and LUSC. (B) Survival curves for OSR2 high and low patients in LUAD from GEO datasets.


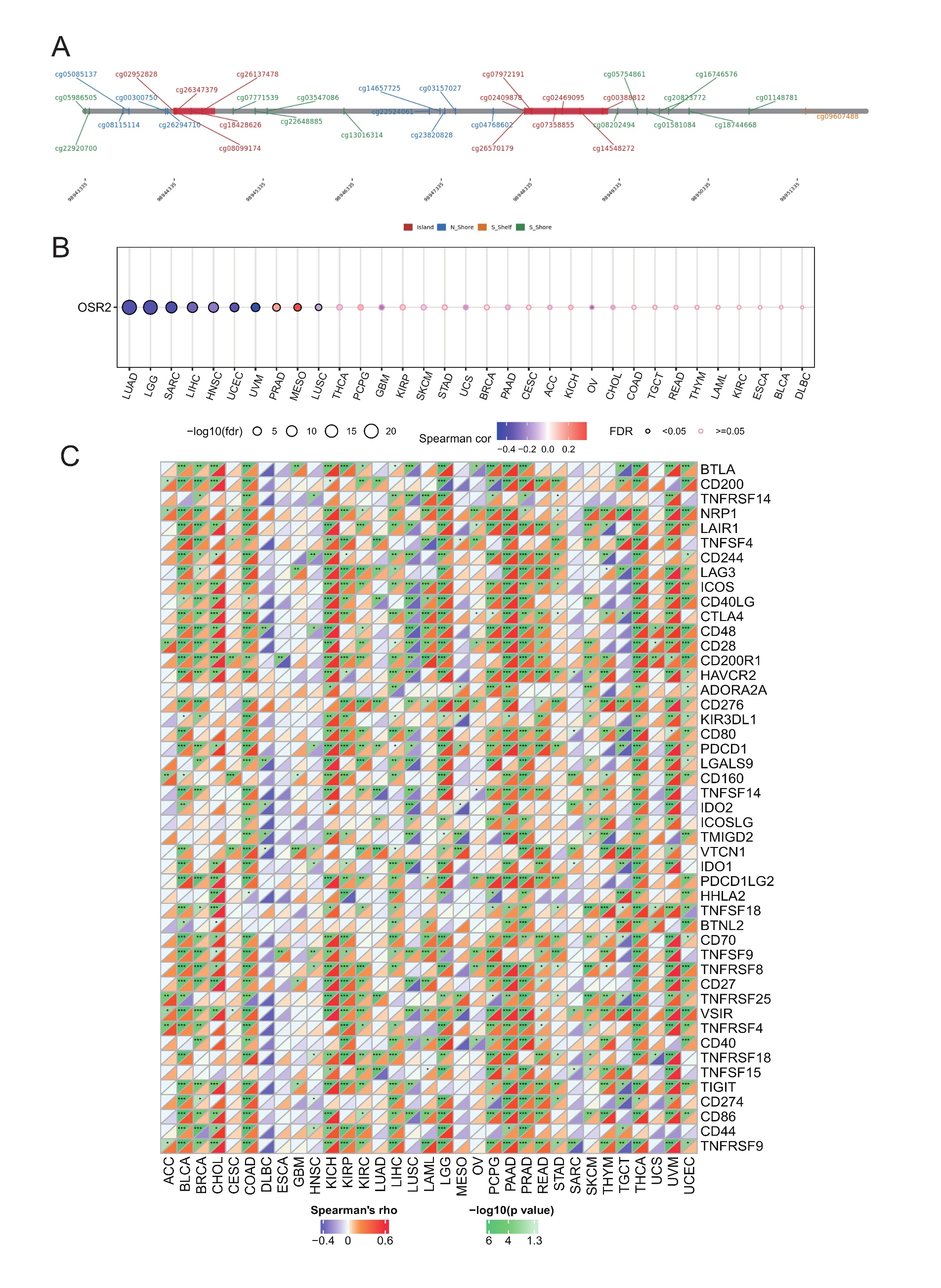


Supplementary Fig.2 (A) Specific CpG probes of OSR2 and their genomic locations. (B)Correlation between OSR2 expression and DNA methylation from GSCA database. (C) Correlation of OSR2 expression and immunomodulatory gene expression in pan-cancer.


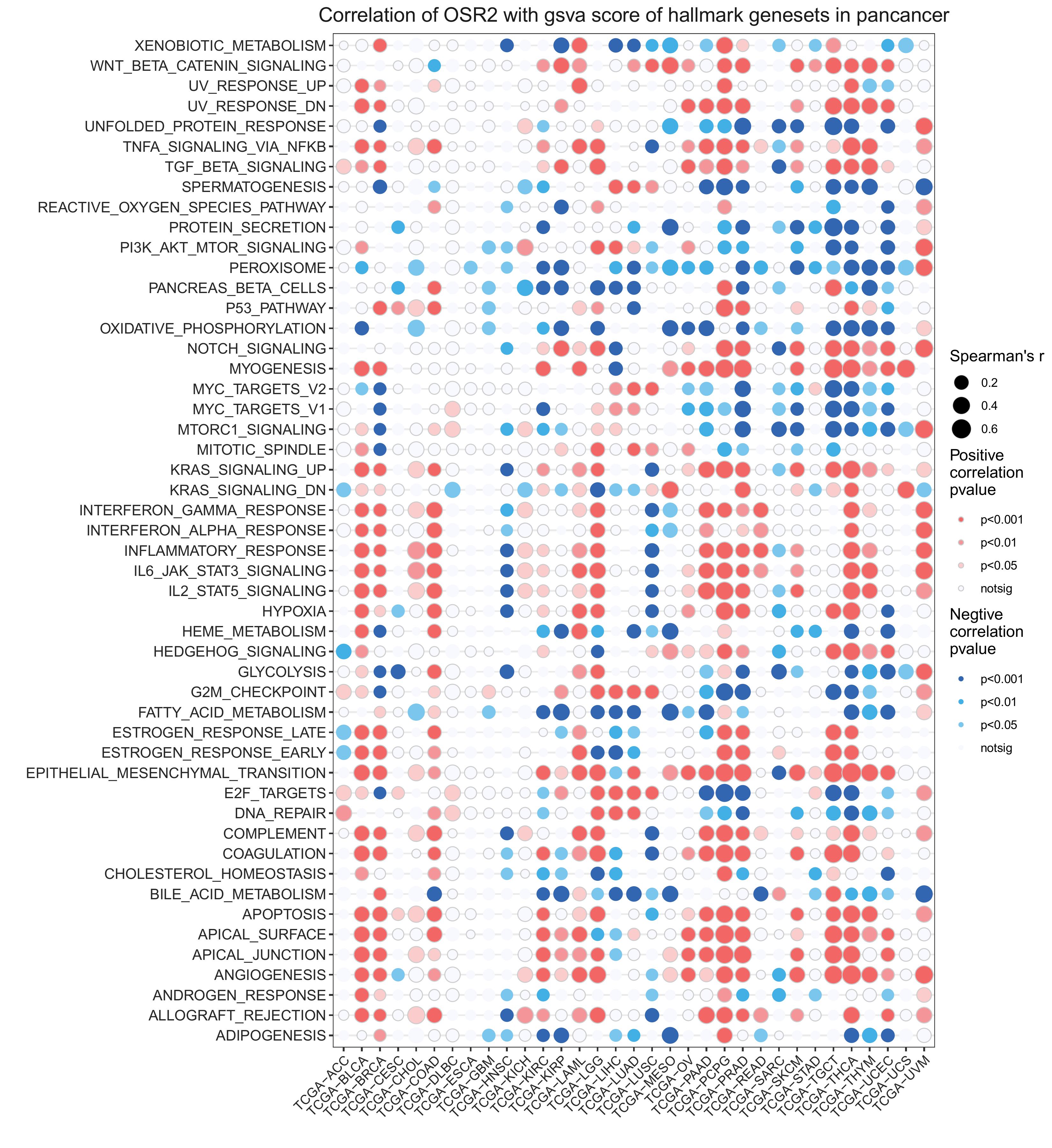


Supplementary Fig.3 Correlation of OSR2 expression with GSVA scores of hallmark pathways in pan-cancer analysis.


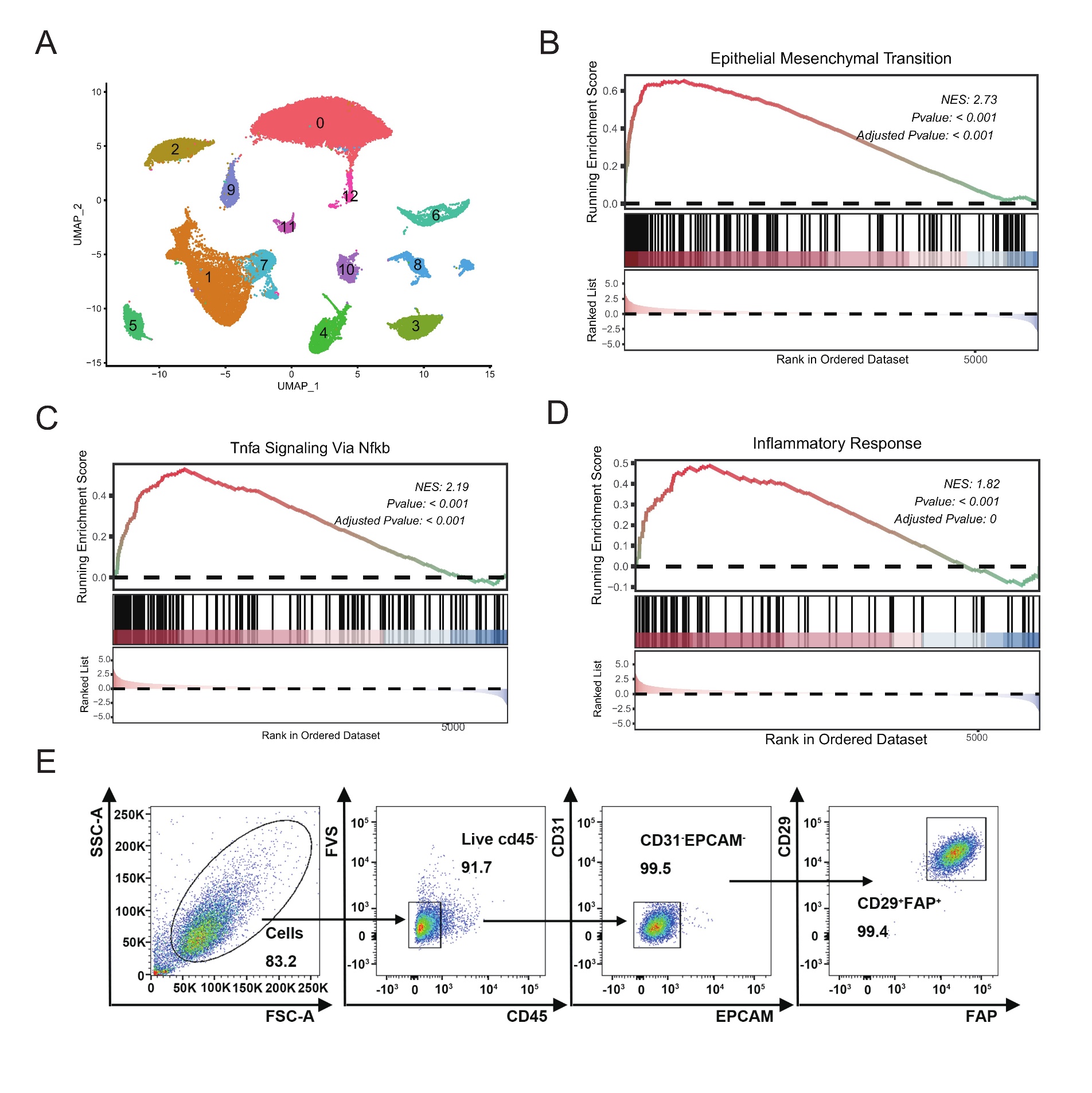


Supplementary Fig.4 (A)Resolution parameter set to 0.1 in clustering analysis to identify distinct cell clusters, visualized in a scatter plot demonstrating the segregation of cellular subgroups. (B-D) GSEA reveals significant enrichment of inflammatory response, EMT and TNFA pathways in the OSR2-high C1_CAF subtype compared to other CAFs. (E) Flow cytometric gating strategy and purity assessment of isolated CAFs.
